# Adaptive Advantage of Myrmecochory in the Ant-Dispersed Herb *Lamium amplexicaule* (Lamiaceae): Predation Avoidance through the Deterrence of Post-Dispersal Seed Predators

**DOI:** 10.1371/journal.pone.0133677

**Published:** 2015-07-21

**Authors:** Koki Tanaka, Kanako Ogata, Hiromi Mukai, Akira Yamawo, Makoto Tokuda

**Affiliations:** 1 Faculty of Agriculture, Saga University, Saga, Japan; 2 The United Graduate School of Agricultural Sciences, Kagoshima University, Kagoshima, Japan; 3 Forestry and Forest Products Research Institute, Ibaraki, Japan; 4 Faculty of Agriculture and Life Science, Hirosaki University, Hirosaki, Japan; Arizona State University, UNITED STATES

## Abstract

Seed dispersal by ants (myrmecochory) is found worldwide, but the benefits that plants obtain from this mutualism remain uncertain. In the present study, we conducted laboratory experiments to demonstrate seed predator avoidance as a benefit of myrmecochory using the annual ant-dispersed herb *Lamium amplexicaule*, the disperser ant *Tetramorium tsushimae*, and the seed predatory burrower bug *Adomerus rotundus*. We compared the predation intensity of *Lamium amplexicaule* seeds by *Adomerus rotundus* under the presence or absence of *Tetramorium tsushimae*. Both the number of seeds sucked by *Adomerus rotundus* adults and the feeding duration of sucked seeds by nymphs were significantly reduced in the presence of ants. This effect was most likely due to the behavioral alteration of *Adomerus rotundus* in response to the ant presence, because ants seldom predated *Adomerus rotundus* during the experiment. Our results demonstrated that the presence of ants decreases post-dispersal seed predation, even when the ants do not bury the seeds. The present study thus suggests that the non-consumptive effects of ants on seed predators benefit myrmecochorous plants.

## Introduction

Seed dispersal by ants, known as myrmecochory, is a common and ecologically important dispersal mode reported over wide geographic areas and in diverse plant taxa [1–3]. Myrmecochorous plants generally have a white or yellowish seed appendage called an elaiosome [3–5], which is rich in nutrients such as lipids, sugars, and amino acids [6]. Ant workers return seeds to their nest, consume the elaiosomes there, and then abandon the unharmed seeds near the nest, thereby contributing to seed dispersal [4]. Other types of ant attractants are also known for several plant species including the odor of an ant-garden epiphyte [7] and the seed coat of an annual weed [8] that similarly function to induce seed dispersal by ants. A recent phylogenetic study revealed that myrmecochory has evolved independently up to 101 times in flowering plants, indicating strong benefits for plants that rely on ants as dispersers [3]. Several hypotheses have been postulated regarding the nature of these benefits, including predator avoidance [9–10], competition avoidance [11–12], and directed dispersal to favorable habitats [1, 3–14]. These hypotheses have been tested by many researchers (reviewed by [4, 15–16]) but remain under active debate [8, 17–19].

The predator avoidance hypothesis states that the selective advantage of myrmecochory lies in the protection of seeds from seed predators [9], although experimental demonstration of this hypothesis remains insufficient [10, 17]. As claimed by [10] and [17], most previous studies have shown that ants remove seeds earlier than do seed predators, but the ultimate fate of these seeds was not clarified [9, 20–21]. Although several studies have demonstrated that seed burial by ants is effective for avoiding seed predators [9–10], ants do not always bury seeds [22–23]. For example, [18] reported that over 90% of seeds removed by ants were relocated to the ground surface after the detachment of elaiosomes. Under such situations, the benefit arising from seed burial would be limited [18]. Therefore, further studies are needed to demonstrate the appropriateness of the predator avoidance hypothesis and especially to reveal the fate of seeds abandoned by ants on the ground around their nests.

Even when seeds are not buried, they can still be protected from predators when placed near ant nests where ant workers are relatively abundant [17]. In a field observation of the tropical myrmecochore *Croton sonderianus* Muell. Arg. (Euphorbiaceae), seed predation rates by insects on the ground were lower for seeds placed near ant nests than for those situated far from the nests, suggesting that a higher density of ants may reduce seed predation rates [17]. In addition, the reduction in the feeding time of seed predators due to direct and indirect interference by ants may also contribute to the increased performance of myrmecochorous seeds, as the loss of endosperm leads to germination failure [24]. Further experiments are needed to confirm these possibilities, as seed predation rates and times near ant nests may be affected not only by high ant density but also by correlated factors, e.g., variations in the habitat preferences of ants and seed predators.

In the present study, we focused on the annual herb *Lamium amplexicaule* L. (Lamiaceae) and an associated burrower bug *Adomerus rotundus* (Hsiao) (Heteroptera: Cydnidae). *Lamium amplexicaule* grows widely across ruderal habitats in Europe and Asia [25] and the seed has an elaiosome and is dispersed by ants [5]. *Adomerus rotundus* is distributed from Hokkaido to Kyushu, Japan; in the Korean Peninsula; China; and the Russian Far East [26] and the burrower bug sucks the seeds of *Lamium* plants [27]. The mother of *A*. *rotundus* produces post-hatch trophic eggs, which are given to hatchlings as their first foods. She then guards the nymphs, frequently leaving the nest to provision them with seeds of the host plant [27, 28]. Nymphs of the late second instar start to forage for seeds independently. During foraging, the burrower bugs may suffer high predation pressure from ants, as has been shown in a closely related species, *Adomerus triguttulus* Motchulsky, also associated with *Lamium* [29].

**Fig 1:**
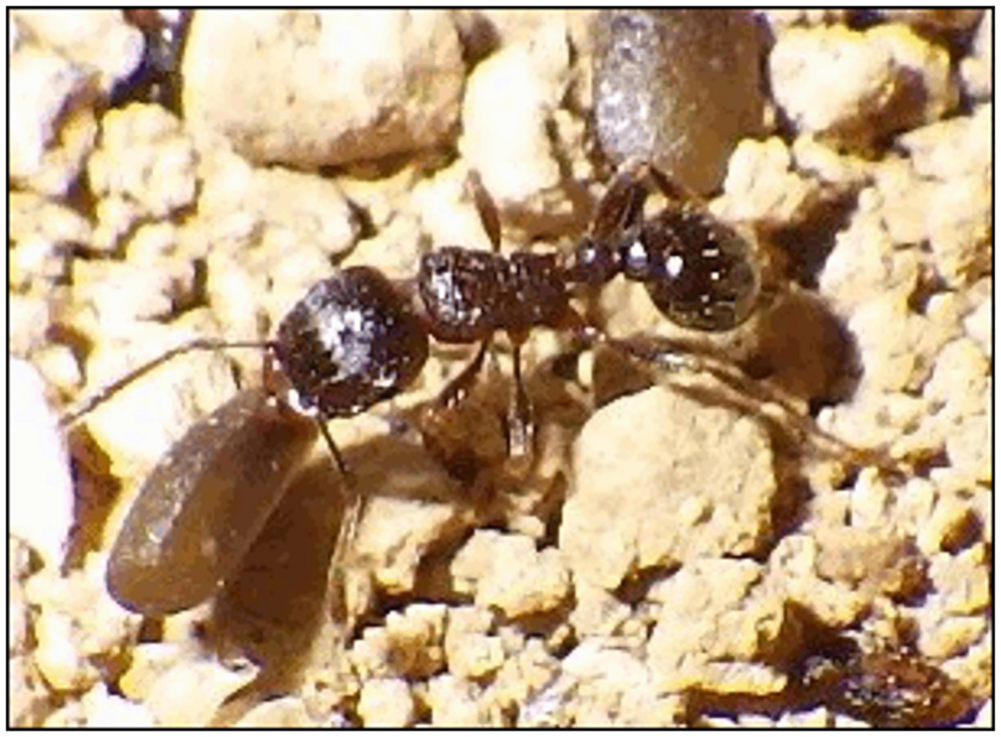
A worker of *Tetramorium tsushimae* transporting a seed of *Lamium amplexicaule* into its nest.

In our census sites, *Tetramorium tsushimae* Emery is frequently observed removing seeds of *L*. *amplexicaule* (Fig 1). Although *T*. *tsushimae* is regarded as a granivore for some plant species [30], our preliminary experiment using two colonies of *T*. *tsushimae* showed that all 60 offered seeds of *L*. *amplexicaule* (30 for each colony) were deposited outside of the nests uninjured, after detachment of the elaiosomes. A previous study [31] reported that *T*. *tsushimae* workers protect seeds from pre-dispersal seed predators in the dyszoochorous annual herb *Chamaesyce maculata* (L.) Small (Euphorbiaceae) by decreasing the feeding activity of seed predator stinkbugs. Because the seeds removed by *T*. *tsushimae* are often deposited on the ground near the nests as observed in other ant species [17, 22], the ant workers may be effective also against post-dispersal seed predators. For *L*. *amplexicaule*, the protection of seeds is important because fallen seeds are consumed by several species of burrower bugs [27, 29] and carabid beetles [32].

In this study, we conducted laboratory experiments to test the effects of ants on the seed predation rate and feeding time of the burrower bug, *A*. *rotundus*. Specifically, we examined the seed predation rates by *A*. *rotundus* under the presence or absence of *T*. *tsushimae* workers. Then we quantified the damage to seeds sucked by *A*. *rotundus* in terms of the decline in seed mass.

## Materials and Methods

### Study species

Mature seeds of *L*. *amplexicaule* were collected during the period from October 2013 to November 2014 from the campus of Saga University, Saga, Japan (33°24′N; 130°29′E), then kept in a refrigerator at 4°C until used in the experiment. Seeds were collected from several individuals and mixed before use.

*Tetramorium tsushimae* is a small (2–3 mm body length) omnivorous ant distributed in most parts of Japan, i.e., from Hokkaido to Yakushima; China; and the Russian Far East [33]. Five colonies of *T*. *tsushimae* were collected in October 2013 from grasslands in Honjo, Saga, Japan (33°24′N; 130°28′E). For experimental use, each original colony was thinned to one queen, 500 workers, and an unspecified amount of brood by at most one month before the experiment. Each colony was placed in an artificial nest made with a glass test tube (17 cm long), kept in the laboratory at 25°C and a 16L: 8D photoperiod, and fed with two mealworms every three days and sucrose water ad libitum.

The *A*. *rotundus* individuals used in the experiment were derived from a stock population started from adults collected in Chinzei-machi, Karatsu, Japan (33°31′N; 129°53′E) and maintained for five years in the laboratory [27]. All individuals were housed in plastic containers with moist cleaning paper (Kimwipe; Nippon Papar Crecia, Tokyo), and kept under the same condition as the ants. Sufficient seeds of *L*. *amplexicaule* were provided for *A*. *rotundus* approximately every other day. Adults and fifth instar nymphs were used in the experiment because earlier instars exhibit relatively low feeding activity.

### Effects of ants on seed predation by burrower bugs

**Fig 2:**
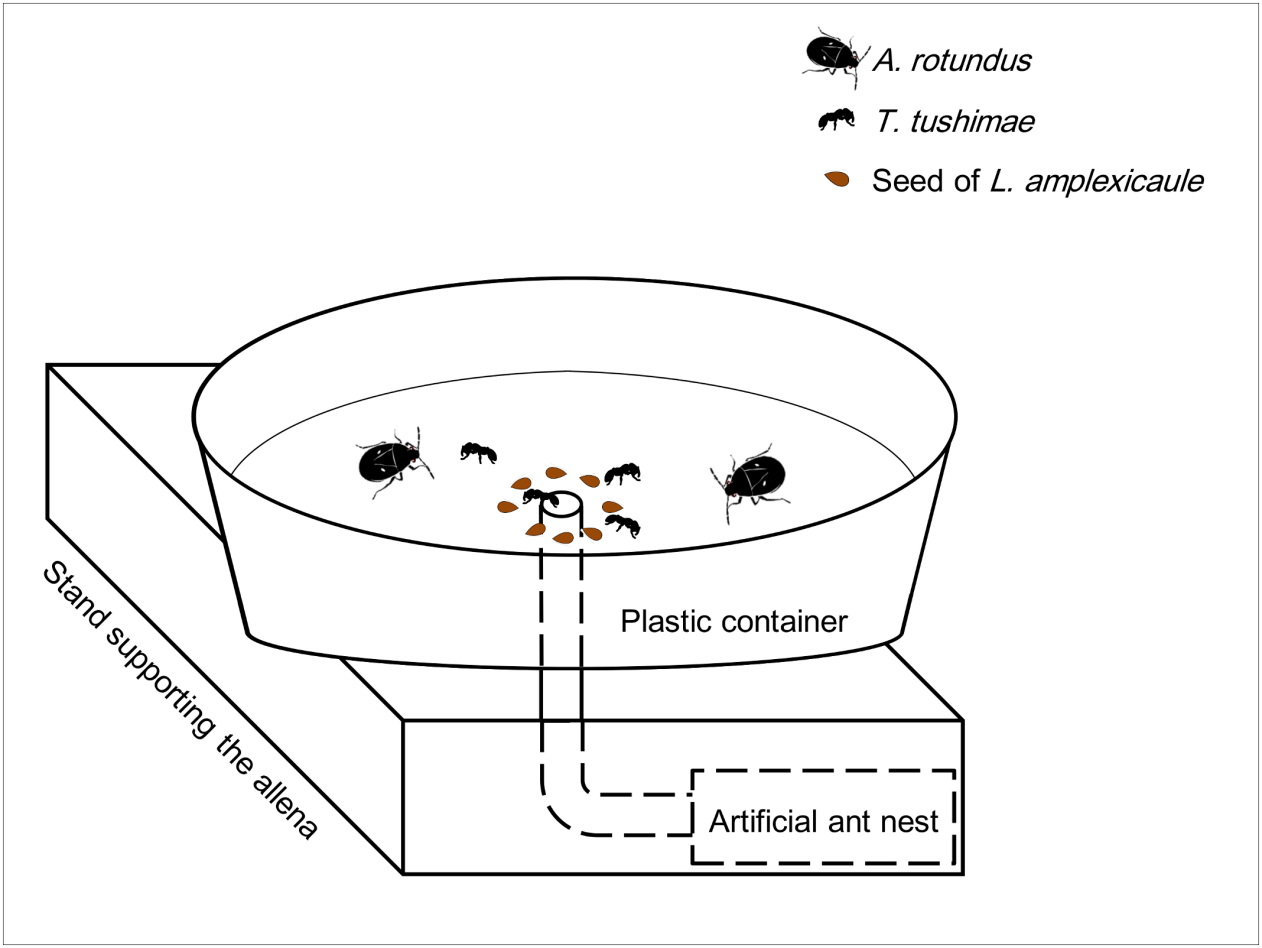
Illustration of the experimental apparatus.

Laboratory experiments were conducted from October 2013 to January 2014 and in December 2014 to demonstrate the role of ant workers in the deterrence of post-dispersal seed predators. White plastic containers (22.5 cm diameter and 7.2 cm deep), the bottoms of which were lined with Kimtowels (Nippon Paper Crecia, Tokyo), were used as the experimental arenas. Three containers were assigned to both the treatment and control, respectively. The inner side walls of the plastic containers were coated with talc powder to prevent the ants and burrower bugs from escaping. In addition, the edge of the Kimtowels was attached to each plastic container with white plastic tape (19 mm wide) to prevent the burrower bugs from hiding in the gaps between the Kimtowels and the container. A hole was made at the center of each container, and a vinyl chloride tube (6 mm inner diameter and 10 cm long) was connected from the bottom side. In the treatment condition, the other end of the tube was connected to an artificial nest (Fig 2). In the control condition, the tube was not connected to an ant nest but was instead plugged with a cotton ball.

Eight seeds of *L*. *amplexicaule* were placed at regular intervals 2 cm from the center of each container. To prevent ants from removing the seeds during the experiment, the elaiosomes of these seeds were detached in advance by offering the seeds to the same ant colonies used in the experiment. As ants usually consumed elaiosomes several days after seed transportation into nests, all retrieved seeds were dried in appearance. Then, either two fifth-instar nymphs or two adults of *A*. *rotundus* were gently released near the side walls of the container. The individuals were released on opposite sides of the container. The burrower bugs had not contacted ants before the experiments, and each individual was used only once to prevent the potential effects of learning. The experimental arena was videotaped for one hour, and the number of seeds sucked by the burrower bugs, time of each feeding event, and ant attacks, including predation, on the burrower bugs were recorded. Burrower bugs were regarded as “predated” by ants if they were transported by worker ants into ant nests. The burrower bugs and ants were fed only water for one and three days, respectively, prior to the experiment. Experiments were conducted between 1000 and 1900 during the light period. In total, 18 treatment trials and 14 control trials were conducted for nymphs, and 13 treatment trials and 16 control trials were conducted for adults. In order to prevent bias owing to overrepresentation of a particular ant colony, each colony was used for an equal number of trials and allocated to each growth stage of burrower bugs as long as possible. Experimental arenas for the treatment condition were not used for the control condition, and vice versa, to prevent frequent disturbance to the ants.

### Effects of feeding duration per sucked seed on the seed mass

In order to quantify the effect of feeding duration per sucked seed on the seed mass, one intact seed was offered to each of 15 adult and 13 fifth-instar burrower bugs. Seeds used in this experiment were the same as those used in the previous experiment and dried at least one day before offering to burrower bugs to further ensure the loss of attractiveness of seeds to ants. The bugs were different individuals from those used in the previous experiment and fed with only water for 24 h before this experiment. In the experiment, each nymph and adult was individually introduced into a glass petri dish (diameter: 45 mm) covered with an approximately 1 cm layer of plaster of Paris and was monitored for its seed sucking activity. When burrower bugs ceased seed sucking, offered seeds were immediately retrieved, dried for three days in 25°C 50% RH, and weighed by an electronic balance (MC5; Sartrius, Goettingen, Germany). The dry mass of 18 intact seeds was also measured as a control.

### Statistical analysis

The effects of treatment and developmental stage of burrower bugs, as well as their interaction on the number of sucked seeds and the feeding duration per sucked seed were analyzed using a generalized linear model (GLM). The model for the number of sucked seeds was initially analyzed assuming Poisson error but subsequently changed to pseudo-poisson error because of over-dispersion [34]. In the case of feeding duration per sucked seed, normal error was assumed. The developmental stage of the burrower bugs (fifth-instar nymphs vs. adults) was considered in order to ascertain whether the ant effects differed with this variable, as has been found in other insects (e.g., [35]). The significance of treatment effect was tested using the F- test [34] and if the treatment × developmental stage interaction was significant, the treatment effect was tested separately for each developmental stage with significance levels adjusted for multiple comparisons according to the Holm method. When a significant treatment effect was detected for a particular growth stage, among-ant-colony variation in the effect was examined by fitting a GLM to the treatment data only. In the model, fixed effects of colony identity on the respective response variables were tested by F-test. In addition, to deal with the possibility that significant results stem from a particular colony having an exceptionally strong deterrent effect on burrower bugs one ant colony was successively removed from the data and in each time the effect of treatment was tested. The effect of feeding duration per sucked seed on the seed mass was quantified for nymphs and adults separately by GLM assuming normal errors. Seed mass was the response variable, and the feeding duration per sucked seed was included as an explanatory variable. The mass of control seeds were included as data in which feeding duration was zero. Significance of feeding duration per sucked seed was tested by F-test. Log- and identity-link functions were used for the quasi-poisson models and models with normal errors, respectively.

All of the analyses were conducted using R version 3.0.2 [36].

### Ethical statement

No specific permit was required for our study species and we did not deal with endangered or legally protected species. Similarly, no specific permissions were required for our ant collection site because the site is unprotected.

## Results

**Fig 3:**
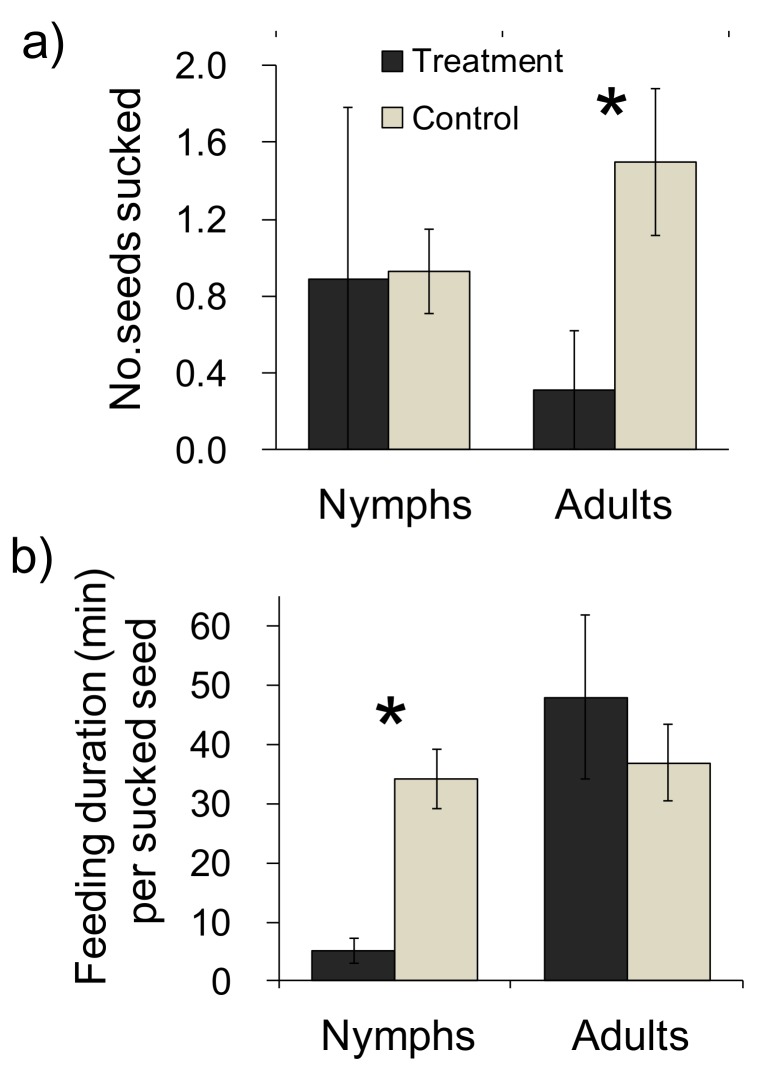
Effects of treatments on (a) the sum of the number of seeds sucked by two burrower bugs (left to right; n = 18, 14, 13, 16 trials) and (b) their feeding duration per sucked seed per two burrower bugs per hour (left to right; n = 7, 9, 4, 12 trials). Black and grey bars denote the treatment and control, respectively, and significant differences between them are denoted by asterisks. Bars indicate the standard errors.

A significant effect of treatment × developmental stage interaction on the number of seeds sucked was detected (*F*_1, 57_ = 4.27, *P* = 0.043), which indicates the differential effect of ant presence on each developmental stage of burrower bugs. Separate analyses for each developmental stage showed that the number of sucked seeds was significantly reduced by ant presence in adults (*F*_1, 27_ = 10.68, *P* < 0.01), but not in nymphs (*F*_1, 30_ = 0.01, *P* = 0.93, Fig 3a). Significant interaction between treatments and developmental stages was also detected for the feeding duration per sucked seed (*F*_1, 28_ = 7.81, *P* < 0.01). Contrary to the number of sucked seeds, the treatment effect was significant in nymphs (*F*_1, 14_ = 22.99, *P* < 0.01, Fig 3b) and not significant in adults (*F*_1, 14_ = 0.67, *P* = 0.43, Fig 3b). In fact, nymphs in ant treatment quite often stopped feeding after sucking a seed for only five minutes (mean ± SE: 5.16 ± 2.15, n = 7). A significant effect of colony identity was detected for the number of seeds sucked by adult burrower bugs (*F*_4, 8_ = 16.09, *P* < 0.01) but not for the feeding duration of nymphal burrower bugs (*F*_4, 13_ = 2.75, *P* = 0.07). Successive removal of one ant colony never altered the significance of the results, indicating that the significant treatment effect was not caused by a particular ant colony having exceptional deterrent effects on burrower bugs.

Although ants attacked burrower bugs in 81% (25/31) of treatment trials, they predated bugs only in seven cases (23%). Indeed, even excluding the data of events in which ants predated the burrower bugs did not alter the results (number of sucked seeds in adults; *F*_1, 23_ = 5.79, *P* = 0.025, feeding duration per sucked seed; *F*_1, 14_ = 22.99, *P* < 0.01).

**Fig 4:**
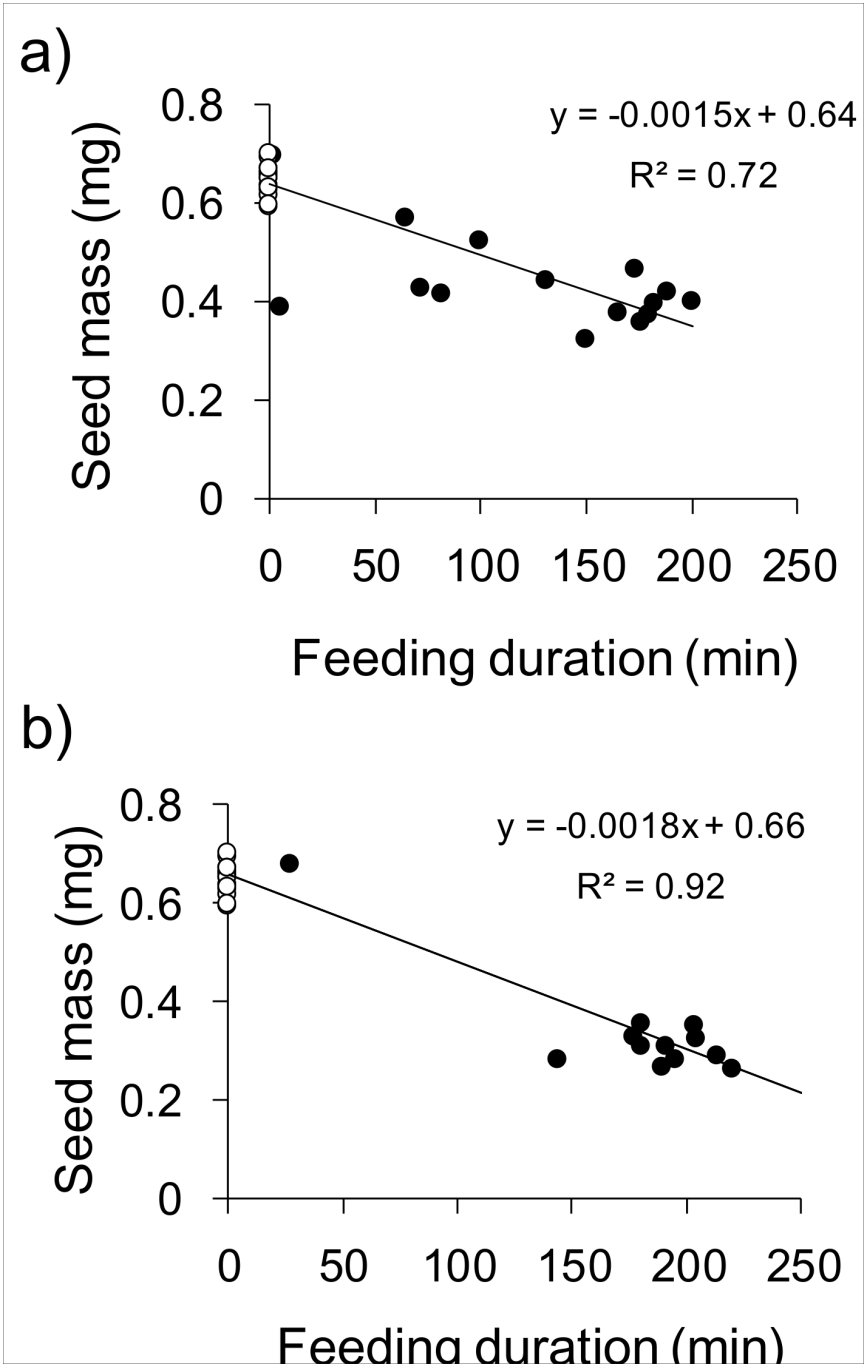
The relationship between feeding duration (min) and seed mass in the seeds sucked by (a) nymphal and (b) adult burrower bugs. The data on intact seeds (n = 18) are shown by the white plots. Note that the same data are used for the intact seeds in both (a) and (b).

The mean weight of intact *L*. *amplexicaule* seeds was 0.66 ± 0.01 (SE) mg (n = 18). Longer feeding duration resulted in more reduction in the seed mass in both nymphs (*F*_1, 31_ = 78.04, R^2^ = 0.72, *P* < 0.01, Fig 4a) and adults (*F*_1, 29_ = 334.9, R^2^ = 0.92, *P* < 0.01, Fig 4b).

## Discussion

The strongest evidence to date supporting the predator avoidance hypothesis has been from studies demonstrating the effects of seed burial on the avoidance of seed predation [9–10]. However, these examples are sometimes criticized because ants do not always bury seeds [18, 22–23]. A recent study reported a lower predation rate for seeds placed near ant nests, suggesting that seeds on the ground can also be protected by ant workers [17]. In the present study, we experimentally demonstrated that the presence of ants actually reduced the seed predation rates by burrower bugs (Fig 3). Specifically, ant presence reduced the number of seeds sucked by adult burrower bugs per hour (Fig 3a). Although burrower-bug nymphs did not show reduction in the number of seeds sucked per hour, their feeding duration per sucked seed was reduced to almost one-sixth of that seen in the absence of ants (Fig 3b). These results indicate that seed sucking by both adults and nymphs were strongly inhibited by ants. Consistent with a previous study [37], seed sucking by burrower bugs reduced seed mass in a time-dependent manner (Fig 4). Because seed mass is positively correlated with the vigor of seedlings [38–40], its reduction is harmful for the fitness of maternal plants [38]. Therefore, the deterrence of post-dispersal predators by disperser ants is regarded as beneficial for *L*. *amplexicaule*. Our results support the predator avoidance hypothesis from the viewpoint proposed by [17], and suggests that the ant-mediated deterrence of post-dispersal seed predators is important for myrmecochorous plants in general.

Mutualistic ants can reduce herbivory through the direct reduction of herbivore density (consumptive or density-mediated effect) or through changes in the behavioral traits of herbivores (non-consumptive or trait-mediated effect) [41–42]. In the present case, seed protection was fundamentally mediated by a non-consumptive effect, because ant predation on burrower bugs was not observed in most trials and the exclusion of trials with predation from the analysis did not affect the results. Although detailed mechanisms are unclear, two possibilities may explain the behavioral alteration of the burrower bug. First, contact experiences with ants before the bugs encounter seeds may affect feeding behavior, e.g. cause a loss of appetite due to “sustained psychological stress” (*sensu* [43]). Second, the burrower bugs may recognize the presence of ants by detecting visual or chemical cues and alter foraging behavior accordingly. Such behavioral alterations in response to ant cues have been observed for a wide variety of insects that suffer high predation pressure from ants, including Lepidoptera [35, 42], Coleoptera [44], and Diptera [45]. For example, a previous study [44] showed that the chrysomelid beetle *Rhyparida wallacei* avoids host leaves exposed to ant trail pheromone.

In our case, adult and nymph bugs reduced different components of feeding activities in response to ant presence: the number of seeds sucked decreased in adults while the feeding duration decreased in nymphs (Fig 3). For plants, the reduction in seed sucking itself is perhaps more beneficial than that in feeding duration, because even a short contact with burrower bugs can be lethal for seeds if the bugs carry pathogens [46]. The reduction of number of sucked seeds in adult burrower bugs may be involved in the adaptive avoidance of harmful contacts with ants. During the experiment, adult bugs seemed to avoid approaching to the entrance of the ant nest when the frequency of ant attack was high, while such a tendency was not observed in nymphal burrower bugs. The reason why such a response was not observed in nymphs is unclear, but it might be attributed to the premature ability of ant recognition by nymphs. The reduction of feeding duration in nymphs can be more appropriately interpreted as a maladaptive stress response (reviewed in [43]) rather than the adaptive changes in behavior. Such incomplete cognitive abilities in earlier developmental stages can arise either due to insufficient development in the nervous system or the ontogenetic shift in predation pressure (reviewed in [47]). The results of our study also imply that behavioral studies of seed predators are important to understand the effectiveness of ant-mediated seed dispersal in the deterrence of post-dispersal seed predators. In this respect, variation of the deterrent effect among ant colonies detected in this study should be detailed in future to clarify which trait of ant colonies produced the difference in feeding behaviors of burrower bugs.

We demonstrated that myrmecochory is effective for preventing post-dispersal seed predation through the deterrence of seed feeders by ants around their nests. This study is the first report to experimentally show that dispersed seeds are protected even when not buried. However, further studies are needed to quantify the extent of this benefit for plants. To quantify precisely the actual benefit of seed predator avoidance for *L*. *amplexicaule*, the relationship between the feeding time of the burrower bug and the reduction in the seed performance of *L*. *amplexicaule* should be examined in future. Moreover, the role of ants in the avoidance of pre-dispersal predation by burrower bugs should be investigated. As previously mentioned, the reduction of pre-dispersal predation may also be an important benefit of myrmecochory [31]. Because we sometimes observe *A*. *rotundus* and ants on *L*. *amplexicaule*, ants may also play a role in decreasing pre-dispersal seed predation by burrower bugs. The benefit of seed dispersal to ant nests shown in our study would be diminished by the clumping of seeds around ant nests and the subsequent intraspecific competition among seedlings [48–49]. Future clarifications of the mechanism of behavioral alteration in the burrower bug, as well as the fitness consequences of predator avoidance, will promote our understanding of the evolution of myrmecochory in a diverse range of plants.
